# Estimating Costs Associated with Disease Model States Using Generalized Linear Models: A Tutorial

**DOI:** 10.1007/s40273-023-01319-x

**Published:** 2023-11-10

**Authors:** Junwen Zhou, Claire Williams, Mi Jun Keng, Runguo Wu, Borislava Mihaylova

**Affiliations:** 1 Health Economics Research Centre, Nuffield Department of Population Health, University of Oxford, Old Road Campus, Headington, Oxford, OX3 7LF UK; 2 Health Economics and Policy Research Unit, Wolfson Institute of Population Health, Queen Mary University of London, London, UK

## Abstract

Estimates of costs associated with disease states are required to inform decision analytic disease models to evaluate interventions that modify disease trajectory. Increasingly, decision analytic models are developed using patient-level data with a focus on heterogeneity between patients, and there is a demand for costs informing such models to reflect individual patient costs. Statistical models of health care costs need to recognize the specific features of costs data which typically include a large number of zero observations for non-users, and a skewed and heavy right-hand tailed distribution due to a small number of heavy healthcare users. Different methods are available for modelling costs, such as generalized linear models (GLMs), extended estimating equations and latent class approaches. While there are tutorials addressing approaches to decision modelling, there is no practical guidance on the cost estimation to inform such models. Therefore, this tutorial aims to provide a general guidance on estimating healthcare costs associated with disease states in decision analytic models. Specifically, we present a step-by-step guide to how individual participant data can be used to estimate costs over discrete periods for participants with particular characteristics, based on the GLM framework. We focus on the practical aspects of cost modelling from the conceptualization of the research question to the derivation of costs for an individual in particular disease states. We provide a practical example with step-by-step R code illustrating the process of modelling the hospital costs associated with disease states for a cardiovascular disease model.

**Supplementary Information:**

The online version contains supplementary material available at 10.1007/s40273-023-01319-x.

## Key Points for Decision Makers

**Table:** 

Estimates of costs reflecting heterogeneity between individual patients are required to inform patient-level decision analytical models, but practical guidance on their estimation is lacking.
This tutorial provides a step-by-step guide to estimating costs associated with disease states using individual patient data, including dataset preparation, statistical model selection, covariate selection and cost model utilization.
A practical example of estimating hospital costs of cardiovascular disease model states and the corresponding R code further illustrate the process.

## Introduction

Decision analytic disease modelling is a common approach used in health economic evaluations. Decision models typically focus on key disease states, represented by disease events or stages, to project disease trajectory given an individual’s characteristics and risk factor profile at entry. To inform economic evaluations, disease models require estimates of costs associated with the model states. Increasingly, decision analytic models are developed using patient-level data with a focus on heterogeneity between patients [1–4] and there is a demand for costs informing such models to reflect heterogeneity between individuals. For example, an economic evaluation of a cardiovascular disease (CVD) prevention strategy may employ a microsimulation disease model to project cardiovascular disease trajectory and survival of patients with particular characteristics. For economic evaluation, this model will also need information about the annual costs of these patients in each disease model state (e.g. with and without cardiovascular event or in year of death). These costs may differ between men and women and depending on their age, lifestyle or previous morbidities. Therefore, cost models are employed to estimate the costs associated with disease model states, taking into account such individual characteristics [1, 5–8].

Models of healthcare costs need to recognize the specific features of costs data, which typically include a large number of zero observations for non-users, and a skewed and heavy right-hand tailed distribution due to a small number of heavy healthcare users [9]. Different methods are available for modelling costs, such as generalized linear models (GLMs), extended estimating equations and latent class approaches [9]. GLMs address the issue of linearity between the linear predictor and the dependent variable and accommodate the skewness in the distribution of the residual error by fitting a link function between the linear predictor and outcome and a variance function. Extended estimating equations extend the GLM by providing more flexibility for the link and variance functions, but require larger samples for the estimation. Latent class approaches assume each individual belongs to one of a set of latent classes, with each class having its own density function contributing to the overall density function. The approach leads to more robust estimates, but its use has been limited by computational complexity and inability to accommodate well excess zeros. Generally, in modelling cost data, it is recommended to use simple methods when having large datasets, and address the small number of key data issue with smaller datasets [10].

Previous tutorials have addressed approaches to disease modelling [11, 12]. However, there is no such guide on how to estimate costs associated with disease model states. Although there are textbooks available for modelling healthcare costs [9, 13], they are not specific for generating costs evidence for supporting health economic models and evaluations. It is still habitual among researchers to conduct economic evaluations using published costs or crude estimates of costs associated with disease model states (e.g. average costs). Therefore, we aimed to present a general approach for the modelling of costs of disease model states using individual participant data. In this tutorial, we focus on the practical aspects of cost modelling from conceptualizing the research question to the derivation of costs for an individual in particular disease states. Specifically, we present a step-by-step guide to how individual participant data can be used to estimate costs over discrete periods for participants with particular characteristics, based on the GLM framework [9]. However, the concepts and steps of cost modelling are applicable regardless of the particular statistical method chosen and readers are advised to explore different methods used for modelling cost data [9, 10].

## Statistical Modelling of Costs Associated with Disease States

To inform decision analytic disease models with the cost evidence, our research question is what are the costs associated with disease states over discrete time periods corresponding to the cycle length of a decision model. The costs can be any type of costs such as total healthcare costs (for example, primary and/or hospital care costs), patient out-of-pocket costs or social care costs. The disease states are key states related to the disease and/or intervention, which are included in the decision analytic model to assess the cost effectiveness of the intervention. For example, disease states may be disease stages or events, such as cancer progression stages or whether experiencing a myocardial infarction (MI). The scope of costs, disease states of interest and cycle length should be consistent with the choices made while conceptualizing the economic evaluation and decision model. In addition, key patient characteristics may also be important factors in the economic evaluation and thus in developing the estimation of costs of disease states using participant-level data, since they may modify health effects, costs and possibly the cost effectiveness of the intervention.

To answer the research question, we will ideally use a longitudinal dataset from a cohort of participants reporting their healthcare and other resource use and costs and disease status over time. This longitudinal data will be used to form estimation data, which have multiple records per participant with each record including the costs accumulated over the periods of interest and the disease state status in the respective periods. All the records from all the participants will be pooled to develop the cost prediction model using participants’ profiles and time-updated disease state status. The developed cost model will allow the prediction of individual patient costs, taking into account participant characteristics, model states and the interactions between them.

### Step 1. Preparing the Dataset for Estimating Costs of Disease States

#### Raw Dataset Generation

The first step is to prepare the dataset to support the cost estimation analysis. This dataset should include records for each participant for discrete time periods over which costs are estimated, with each record including the outcome cost variable and a number of covariates representing the participant’s characteristics. For example, if data is available for the hospital care costs of an individual over 10 years but we are interested in estimating annual hospital care costs, we would allocate costs into respective annual periods in chronological order and generate 10 records or rows with annual costs for this individual. Each row represents a unique record contributing to the analysis. The column of costs over the discrete periods (e.g. annual hospital care cost) represents the outcome.

**Fig. 1:**
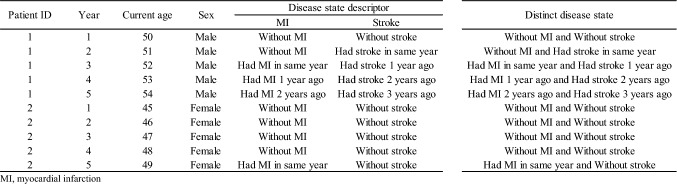
Schematic of dataset for modelling healthcare costs associated with disease states

Two types of individual characteristics are further needed to estimate costs of disease states: the disease states’ indicators and the other individual characteristics associated with the costs. The disease states’ indicators are specific to the individual and each discrete period of time but can change across time periods with an individual’s disease trajectory. For example, an individual remains in the ‘without MI’ state until they experience an MI, and move into an annual period ‘had MI in same year’, followed by ‘had MI 1 year ago’ etcetera, corresponding to timing of the MI with respect to the current time period. In this example, ‘without MI’, ‘had MI in same year’ and ‘had MI 1 year ago’ represent different states and, therefore, distinct columns in the dataset to support estimation of costs. Distinct disease states could be specified by more than one disease state descriptor (e.g. ‘without MI or stroke’ requires both ‘without MI’ and ‘without stroke’ descriptors to be met) (Fig. 1). The choice of disease state descriptors is pre-specified but could be adjusted (e.g. number of temporal categories) alongside covariate selection in the model selection step (see step 3). The other individual characteristics of interest include, for example, individual’s age, sex, and other socio-demographic and clinical risk factors that determine the extent of healthcare costs. Ideally, characteristics which are plausible predictors of healthcare costs given the data availability should be prospectively identified prior to cost modelling from previous evidence. Most of these characteristics are likely to be specific to individuals and fixed at entry into the model but some, such as age, may be updated over the time periods in the dataset.

#### Handling Censored and Missing Data

Typically, individual patient data is subject to administrative censoring (e.g. end of data collection due to end of follow-up in the study). In our context, death is an event of interest and not a censoring event; all costs in year of death are observed. In effect, ‘death in year’ is usually a covariate in the cost model as we want to assess its impact on costs. Simple approaches to handling censored cost data are to (1) add a covariate indicating the proportion of period unobserved; or (2) exclude all observations with partially observed data due to censoring (if sample size is generous).

We may encounter missing costs data, frequently the case when costs data is collected from the patients (e.g. case report form in a clinical trial) rather than sourced from linked routine healthcare data (e.g. hospital or primary care data). Generally, multiple imputation under the missing-at-random assumption is used in this context as single imputation methods overstate precision [10]. Violations of the missing-at-random assumption, a particular consideration in the presence of substantial attrition in the sample, would require further methods [14–16]. Besides, we may also need to handle missing values of covariates, which has been discussed in detail elsewhere [17].

#### Covariate Specification

For continuous covariates, we will need to specify their functional form in the model. If the relationship between the covariate and the outcome is known, we can transform the covariate correspondingly (e.g. natural logarithm transformation). Such a relationship can be informed from previous studies or preliminary analyses. When the relationship is complex, other approaches, including (1) specifying spline effects; (2) specifying polynomial effects and (3) categorization [16] should be considered.

To facilitate model interpretation, we recommend standardizing continuous covariates and for discrete (binary and categorical) covariates to have an explicit choice of reference category. For example, for a cohort with mean and standard deviance of age of 59 and 9 years, respectively, we can standardize age by centring at 60 years, a round number close to mean, and expressing it per 10 years using a transformation: (age—60)/10; for BMI (kg/m^2^) categorized into underweight (< 18.5), healthy weight (18.5–25), overweight (25–30) and obesity (≥ 30), we can choose the healthy BMI as the reference category.

### Step 2. Candidate Statistical Models for Estimating Costs of Disease States

#### Common Candidate Statistical Models

The statistical models for modelling costs are chosen based on the features of cost data and the features of statistical models. A feature of cost data is that the distribution of the costs is typically right skewed (long tail at the higher costs), which may not be suitable for ordinary linear regression that requires normality and homoscedasticity in the residuals (i.e. error). Therefore, the GLM framework is often employed by specifying a link function $g$ and family distribution, which standardize the mean and variance function. Through the inverse link function $({g}^{-1}\left(.\right))$, $E\left(y|x\right)=\mu$, the expected value of the cost *y* given a vector of covariates *x*, can be calculated from the linear predictor $(x\beta )$:

$$g\left(\mu \right)=x\beta$$

$$\mu ={g}^{-1}(x\beta )$$

where $\beta$ is the vector of the regression coefficients.

In a GLM, $$\mu \propto v\left(y|x\right)={\theta }_{1}{\mu }^{{\theta }_{2}}$$

where $\mu$, $y$ and $x$ are as above, $v$ is the variance, ${\theta }_{1}$ is a constant, and ${\theta }_{2}$ indicates the mean–variance power relationship.

${\theta }_{2}=0$ corresponds to a Gaussian error variance, ${\theta }_{2}=1$ to a Poisson variance, and ${\theta }_{2}=2$ to a Gamma variance.

For modelling healthcare costs, three common distributions are Gaussian, Poisson and Gamma distribution. Depending on the distribution, common link functions are identity, natural logarithm, inverse and the squared root link. The most popular ones (combinations of link function and distribution) for healthcare costs are linear regression (identity link with Gaussian distribution) and Gamma regression with a natural logarithm link [9].

Another feature of cost data is a large proportion of zero observations. This is usually addressed using two-part models, with the first part, typically a logistic or probit regression, modelling the probability of incurring any cost, and the second part modelling the cost conditional on incurring any [9]. The expected cost from the two-part model is the product of the expectation of each part:

$$E\left(y|x\right)=Prob\left(y>0|x\right)E\left(y\right|x, y>0)$$

where $y$ is the cost outcome and $x$ is a vector of covariates.

Both a one-part model (i.e. a single regression equation) and two-part model (two regression equations with the first modelling the probability of incurring costs and the second the costs, conditional on incurring any) should be considered. We should use six GLM specifications defined using the combinations of two link functions (identity and natural logarithm link) and three variance functions (Gaussian, Poisson and Gamma distribution) as candidate models for the one-part model and the second part of the two-part model.

#### Initial Set of Covariates

For each candidate model specification, the model should be fit to the data to aid model selection. Initially, the full set of the pre-specified covariates from the prepared dataset could be used in every candidate statistical model. We can also perform covariate selection (will be mentioned in Step 3) for each candidate model before the selection of the promising candidate statistical models in the next step.

#### Tests to Choose Statistical Model Specification

##### The Hosmer-Lemeshow test

The appropriateness of the link function can be tested using the Hosmer-Lemeshow test [9, 18]. The test regresses the residual error $(e)$ on binary indicators for the deciles of the predicted costs $({\widehat{y}}_{D1}\,\mathrm{to }\,{\widehat{y}}_{D10})$, and tests the joint significance of the coefficients, with a significant test indicating an inappropriate link function

$$e\sim {\widehat{y}}_{D1}+{\widehat{y}}_{D2}+{\widehat{y}}_{D3}+{\widehat{y}}_{D4}+{\widehat{y}}_{D5}+{\widehat{y}}_{D6}+{\widehat{y}}_{D7}+{\widehat{y}}_{D8}+{\widehat{y}}_{D9}+{\widehat{y}}_{D10}$$

##### The Pregibon link test

The appropriateness of the link function can also be tested using the Pregibon link test [19]. The test regresses the costs from the data $(y^{\prime})$ on the linear predictor $\left({x^{\prime}}\beta \right)$ and a squared linear predictor ${[\left({x^{\prime}}\beta \right)}^2]$ using an identical GLM specification, with a significant coefficient for the squared linear predictor indicating an inappropriate link function

$$y^{\prime}\sim 1+ {x^{\prime}}\beta +{\left({x^{\prime}}\beta \right)}^2$$

##### The modified Park’s test

The appropriateness of the distribution family can be checked using the modified Park’s test [20]. The test reflects the relationship between the variance and the mean for a specific distribution based on a power function mentioned above for different GLM distributions. The modified Park’s test regresses the natural logarithm of the squared residual error ${(\mathrm{ln}(\left(y^{\prime}-\widehat{y}\right)}^2))$ on the natural logarithm of predicted costs $(\mathrm{ln}\left(\widehat{y}\right))$ using a GLM specification with gamma distribution and usually a natural logarithm link. The coefficient close to 0 indicates Gaussian distribution, 1 indicates Poisson distribution, and 2 indicates Gamma distribution

$${\mathrm{ln}(\left(y^{\prime}-\widehat{y}\right)}^2)\sim \mathrm{ln}\left(\widehat{y}\right)$$

Statistical models that demonstrate promise are taken forward.

### Step 3. Selecting the Final Model

The model selection thereafter has two parts: selection of covariates for each remaining candidate statistical model and selection of the statistical model from the final specifications of all candidate statistical models.

#### Covariate Selection

The cost models are intended to predict costs in decision models for patients with particular characteristics at entry. Therefore, cost models should perform well not only across the population but potentially also at the individual patient level. Thus, all covariates retained in models should be reliably associated with cost. To minimize the likelihood of spurious associations, the covariates in final cost models, unless their inclusion was informed from strong previous evidence with consistent estimates in our dataset, are expected to reach statistical significance and their inclusion and retention subject to covariate selection.

Stepwise selection using a pre-specified level of statistical significance (e.g. 5%) is widely used given its simplicity and availability in statistical software [21, 22]. However, the stepwise approaches may lead to unstable selection and an overfitting issue. Alternative covariate selection approaches aiming to address these issues, such as bootstrapping stepwise selection and penalised techniques (e.g. least angle selection and shrinkage operator, LASSO) have been proposed [15]. The bootstrapping approach is an extension of the stepwise approach by performing selection in the bootstrap samples and selecting the covariates based on their frequency of being selected. It has the potential to address the issue of instability of the selection, but has much higher computation burden. The LASSO method constrains the regression coefficients and shrinks some regression coefficient estimates to zero to optimize covariate selection. This approach may address the issue of overfitting, but it may also end up including implausible covariates or omitting known predictive factors [15].

For a two-part model, covariate selection could be performed for each part of the model, as covariates may have different impacts on the probability of incurring the costs and the costs conditional on any incurring.

#### Final Model Selection

Finally, the performance of each final statistical model specification should be checked against the observed costs. The model performance can be assessed with three measures: mean error, mean absolute error, and root mean squared error. Mean error (ME) is the mean of the residual errors, which tests for aggregate bias. Mean absolute error (MAE) is the mean of the absolute value of the residual errors, which tests for individual level predictive accuracy. Root mean squared error (RMSE) is the squared root of the mean of the squared of the residual errors, which tests for goodness of fit. Smaller values for these measures indicate better performing models.

$$ME=Mean(e)$$

$$MAE=Mean(|e|)$$

$$RSME=\sqrt{Mean\left({e}^2\right)}$$

We can also perform a visual inspection of model performance by plotting mean predictive error by decile of predicted outcome to check for systematic errors not detected by ME/MAE/RSME above. Better fitting models have smaller errors across deciles of predicted outcomes.

#### Consideration of Interactions

We can further refine the cost model by considering interactions between key covariates. Such considerations should be pre-specified to limit data dredging. For the cost model of interest, we focus on the interactions between acute disease events (e.g. experiencing MI and stroke in the same year). The overall impact of co-occurring acute disease events on costs may not be a simple addition of the impact of each event. However, it is also difficult to assess all possible interactions in view of the number of possible combinations. We suggest a practical criteria for the choice of interactions to consider based on (1) the number co-occurrences in the same period and (2) the percentage of occurrences from the total individual occurrences for the respective events. The purpose is to assure sufficient data is available to reliably estimate interactions. For example, we may investigate the interaction between MI and stroke if (1) the number of cases when both MI and stroke occur in the same year is more than 50; and (2) both percentages of this number from the total number of MIs and strokes are > 5%. The thresholds may be smaller if we focus on rarer but costly events. Besides, we may also need to consider the interaction between other participant characteristics, which has been discussed in detail elsewhere [16].

### Step 4. Use of the Cost Model

The final cost model can be used to (1) predict the cost for individuals, and (2) derive the mean effects of events on costs across particular patient population/s.

#### Cost Prediction Given Individual’s Characteristics

To predict costs of an individual in a particular time period, we should prepare the individual’s characteristics to correspond to respective characteristics in the model’s specification. Thereafter, for one-part models, we can use the prepared individual’s characteristics together with the model’s parameter estimates to generate the predicted cost. For two-part models, we should use the prepared individual’s characteristics together with parameter estimates of each part of the model, with the first part generating the probability of incurring any costs $(Probabilit{y}_{P1})$ and with the second part generating the costs conditional on incurring any costs $(Cos{t}_{P2})$. With the predictions from both parts, we can generate the predicted costs with the following formula:

$$Predicted\, costs=Probabilit{y}_{P1}\times Cos{t}_{P2}$$

If logistic regression is used for the first part of the two-part model, *Probability*_*P1*_ can be calculated with the odds of incurring any costs $(Odd{s}_{P1})$ from the logistic regression using the following formula:

$$Probabilit{y}_{P1}=Odd{s}_{P1}/(1+Odd{s}_{P1})$$

#### Effect of a Disease State on Costs

Entry into a disease state is often associated with a change in healthcare costs. Cost models can inform changes in healthcare costs associated with a disease state by calculating the marginal effect of disease states in the cost model. For a one-part model with identity link, the marginal effect is represented by the corresponding coefficient in the cost model. For a one-part non-linear model or a two-part model, marginal effects can be derived using recycled prediction. It includes the following two steps: (1) run two scenarios across the target population by setting the disease state of interest to be (a) present (e.g. recurrent cancer) or (b) absent (e.g. no cancer recurrence); (2) calculate the difference in mean costs between the two scenarios. Standard errors of the mean difference can be estimated using bootstrapping.

## Illustrative Example: Modelling Hospital Costs Associated with Cardiovascular Events

We will illustrate the modelling process by taking readers through an exercise of modelling hospital costs associated with cardiovascular events in the UK using individual patient data. The original analyses [8] used the data from the 500,000-large UK Biobank, with rich participant baseline data and linked data from national databases on hospital admissions, cancers and deaths. UK reference costs were used to cost hospital episodes [23] with costs inflated to year 2020 using the NHS cost inflation index [24].

**Fig. 2:**
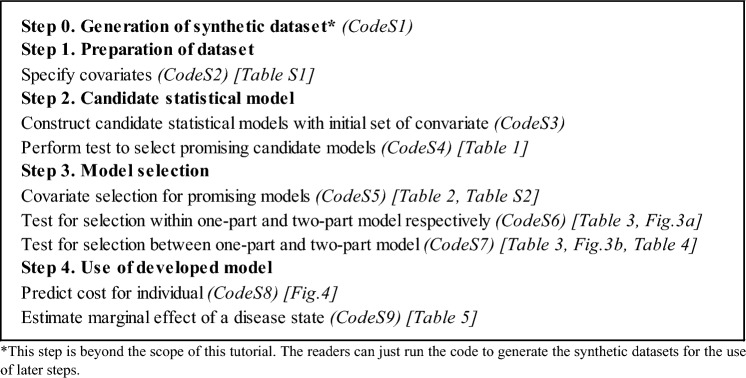
Summary of the steps for modelling healthcare costs associated with disease states in the illustrative example

For the purpose of this tutorial, we focus on modelling annual hospital care costs of people without previous CVD. We created a synthetic analytical dataset with 10,000 participants having 10 annual periods for each participant based on the summary data from the published study [8] for the illustration (Supplementary Section 1, see electronic supplementary material [ESM]). Figure 2 shows the summary of the steps for modelling healthcare costs associated disease states with the illustrative example.

### Step 1. Preparation of Dataset

As the focus is on estimating annual costs associated with disease states, annual periods from entry into the study were formed containing the respective annual hospital care costs. The disease events of interest were incident MI, stroke, vascular death and non-vascular death. Each event except death was specified using a categorical variable with values of ‘without event’, ‘year of event’, ‘year following event’, ‘two years following event’, and ‘three or more years following event’; vascular death and non-vascular death were binary variables indicating whether there was such a death in the year.

Further candidate covariates included age, sex, ethnicity, quintile of Townsend deprivation index, smoking status, physical activity, diet quality, body mass index (BMI), low density lipoprotein (LDL) cholesterol, high density lipoprotein (HDL) cholesterol, serum creatinine, systolic blood pressure (SBP), diastolic blood pressure (DBP), antihypertensive treatment, history of diabetes, history of cancer and of severe mental illness.

In our example, age and disease status (or state) were updated annually, with disease states updated depending on the timing of the event’s occurrence. All the other covariates remained at their baseline values for the purpose of the intended model (in which only disease progression was modelled). For illustration, we used the same way of specifying baseline covariates as those from the published study (Code S2, Supplementary Table S1, see ESM).

### Step 2. Candidate Statistical Models

We chose both one- and two-part models as candidate statistical models. For the two-part model, we chose six models, all of which had the same first part using a logistic regression modelling the probability of incurring any costs, but a different second part using different GLMs modelling the costs conditional on any incurring. These were the following common GLMs (Distribution–Link): ‘Gaussian–Identity’, ‘Gaussian–Log’, ‘Poisson–Identity’, ‘Poisson–Log’, ‘Gamma–Identity’, ‘Gamma–Log’. For the one-part model, we chose only the ‘Gaussian–Identity’ GLM, since fitting GLMs other than linear regression (Gaussian–Identity GLM) to the data with a high proportion of zero-cost observations requires extra effort in finding initial coefficients to fit the model, which is not the focus of this tutorial. Although there was no process for selecting GLM for the one-part model, the process could be reflected from the process of selecting GLM for the second part of the two-part model.

For ease of illustration, we did not perform covariate selection for each candidate model for parsimonious model construction at this step. Instead, we retained all the covariates in the candidate models (Code S3).

We performed the model specification tests for the six candidate GLMs for the second part of the two-part model (Code S4). The slopes from the modified Park’s test for all the GLMs were close to 2, indicating that the Gamma distribution was the most plausible for the variance function. At the significance level of 5%, almost all the *p* values from the Hosmer-Lemeshow test and from Pregibon’s test were not significant, indicating identity and Log link were both acceptable link functions (Table 1). Therefore, the two-part models using ‘Gamma–Identity’ and ‘Gamma–Log’ GLMs as the second part were the most promising candidate two-part models.

**Table 1:** Model specification tests for the second part of the candidate two-part model

GLM model (Distribution–Link)	Slope from modified Park’s test	*p* value from Hosmer-Lemeshow test	*p* value from Pregibon’s test
Gaussian–Identity	1.97	0.12	0.91
Gaussian–LOG	1.96	0.04	0.74
Poisson–Identity	1.98	0.59	0.91
Poisson–LOG	2.00	0.22	0.35
Gamma–Identity	1.98	0.83	0.99
Gamma–LOG	1.99	0.67	0.45

*GLM* generalized linear model

### Step 3. Model Selection

#### Covariate Selection

Covariate selection was performed for all the promising candidate one-part and two-part models using stepwise backward selection at the 5% significance level (Code S5). Table 2 shows the final selected covariates and the detailed selection process for them.

**Table 2:** Covariate selection results

Model	Two-part model—Part 1		Two-part model—Part 2 GLM				One-part GLM	
	Logistic regression		Gamma–Identity		Gamma–Log		Gaussian–Identity	
Selected covariates								
	Age, sex, prior diabetes, MI, stroke, NVD		Age, sex, systolic blood pressure, MI, stroke, VD, NVD		Age, sex, systolic blood pressure, MI, stroke, VD, NVD		Age, sex, antihypertensive treated, MI, stroke, NVD	
Covariate selection process								
Step	Covariate to be dropped^a^	*p* value	Covariate to be dropped^a^	*p* value	Covariate to be dropped^a^	*p*-Value	Covariate to be dropped^a^	*p* value
1	Severe mental illness	0.96	Diet quality	0.87	Diet quality	0.98	Smoking status	0.93
2	VD	0.95	Diastolic blood pressure	0.81	Diastolic blood pressure	0.93	Severe mental illness	0.87
3	Systolic blood pressure	0.88	Townsend score	0.79	LDL cholesterol	0.90	HDL cholesterol	0.79
4	HDL cholesterol	0.69	LDL cholesterol	0.81	Severe mental illness	0.81	Diet quality	0.73
5	Smoking status	0.67	Severe mental illness	0.72	Townsend score	0.71	Serum creatinine	0.62
6	Diet quality	0.59	Serum creatinine	0.64	Serum creatinine	0.67	Prior cancer	0.48
7	Physical activity	0.55	HDL cholesterol	0.61	HDL cholesterol	0.57	Physical activity	0.46
8	Diastolic blood pressure	0.50	Antihypertensive treated	0.58	Antihypertensive treated	0.44	Diastolic blood pressure	0.42
9	Serum creatinine	0.46	Smoking status	0.48	Smoking status	0.44	Systolic blood pressure	0.39
10	Ethnicity	0.41	Prior diabetes	0.33	Prior diabetes	0.35	LDL cholesterol	0.39
11	LDL cholesterol	0.31	Physical activity	0.22	Physical activity	0.30	Ethnicity	0.39
12	Townsend score	0.30	Ethnicity	0.21	Ethnicity	0.21	Townsend score	0.25
13	Prior cancer	0.14	Body mass index	0.16	Body mass index	0.10	VD	0.14
14	Body mass index	0.09	Prior cancer	0.09	Prior cancer	0.09	Body mass index	0.05
15	Antihypertensive treated	0.06					Prior diabetes	0.06

*GLM* generalized linear model, *HDL* high density lipoprotein, *LDL* low density lipoprotein, *MI* myocardial infarction, *NVD* non-vascular death, *VD* vascular death

^a^At each step, the previous dropped covariates were added back to the model one by one to test whether they should be added back, but in the illustrative example none was added back

However, the stepwise approach may result in selection of unstable predictors. Therefore, we illustrate the use of bootstrapping stepwise selection approach with 60% cut-off criteria (e.g. we retain covariates if selected in > 60% bootstrap samples) for the Gamma-Identity GLM of costs conditional on any being incurred (Supplementary Table S2, CodeS5, see ESM), which excluded systolic blood pressure, a nuisance predictor previously included in this model.

#### Statistical Model Selection Within One-Part Models and Within Two-Part Models

**Fig. 3:**
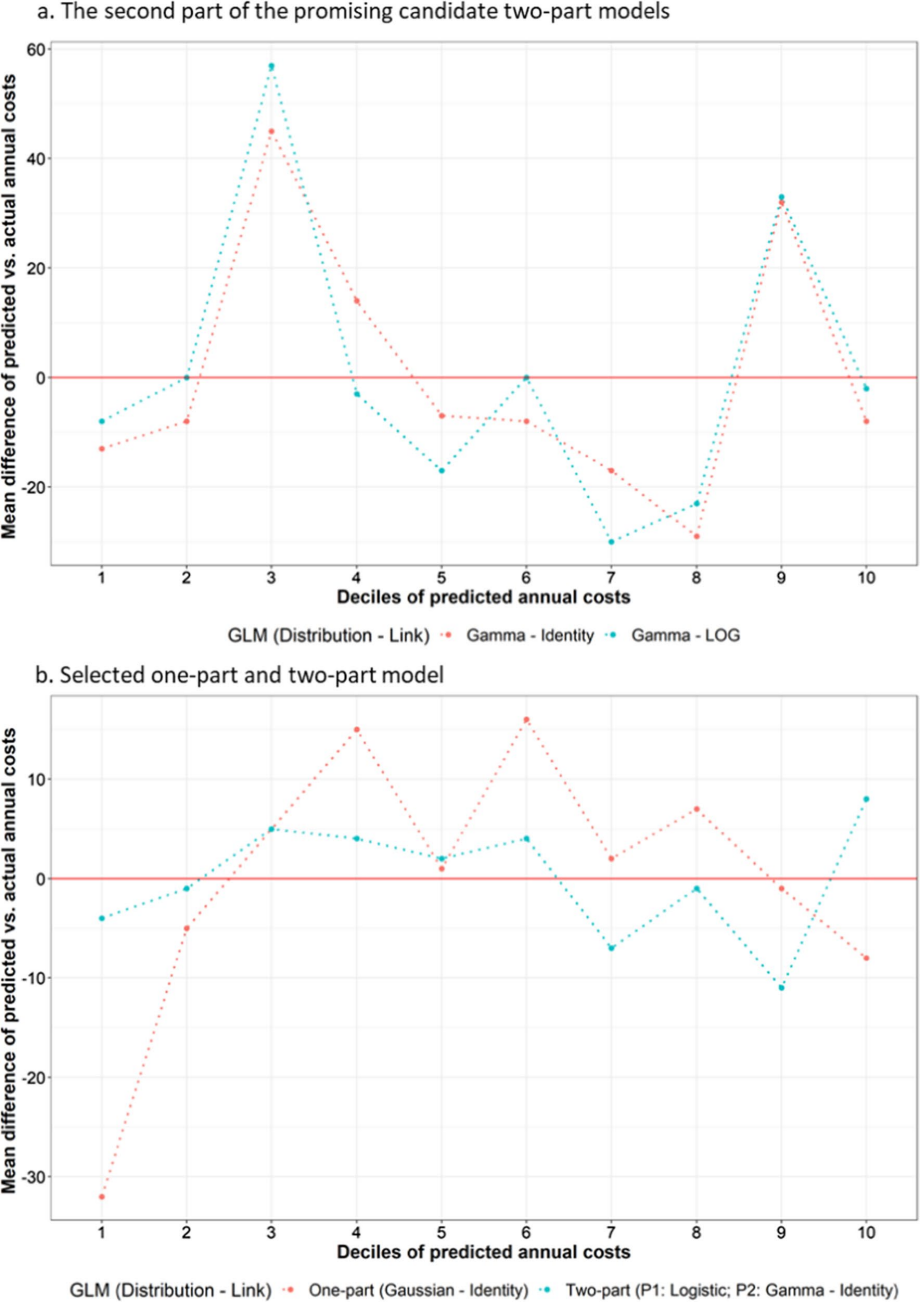
Mean prediction error by decile of predicted costs for candidate model selection. *GLM *generalized linear model

As the ‘Gaussian–Identity’ GLM was the only one-part model we considered, this was the final one-part model. For the two promising candidate two-part models (with the second part using ‘Gamma–Identity’ and ‘Gamma–Log’), further specification tests were performed for the second part of them after covariate selection. The specification tests results were similar to before, with no definitive evidence that one outperformed the other, and model performance tests found similar performance between the two models (Table 3). Plotting the mean prediction error by deciles of predicted costs did not help discern a superior performance either (Fig. 3a). As the GLM using identity link is easier to interpret than those using log link, we selected the two-part model using ‘Gamma–Identity’ GLM as the second part of the final two-part model (Code S6).

**Table 3:** Tests for the promising candidate two-part models and for the selected one-part and two-part models

Candidate model	Model specification test			Model performance test		
	Modified Park's test	Hosmer-Lemeshow test	Pregibon's test	ME	MAE	RSME
Second part of the promising candidate two-part model						
Gamma–Identity	2.00	0.22	0.96	0	856	1115
Gamma–LOG	2.01	0.22	0.39	− 1	856	1122
Selected one-part and two-part models						
One-part using Gaussian–Identity GLM				0	458	826
Two-part (Part 1: logistic regression; Part 2: Gamma–Identity)				0	458	825

*GLM* generalized linear model, *ME* mean error, *MAE* mean absolute error, *RMSE* root mean squared error

#### Statistical Model Selection Between One-Part and Two-Part Models

Model performance tests were conducted for the final one-part and two-part models, and found they had similar performance (Table 3). Plots of the mean prediction error by deciles of predicted costs showed the final two-part model was consistent across most deciles, whereas the final one-part model did not perform well in the first two deciles (Fig. 3b). Therefore, the final two-part model was selected as the final model (Table 4) (Code S7).

**Table 4:** Annual hospital care costs (£) model: two-part model (part 1: logistic regression; part 2: generalized linear model with Gamma distribution and identity link function)

Covariate	Category	Part 1: Probability of incurring cost OR (95% CIs)	Part 2: Cost, if any incurred Mean (95% CIs)
Intercept^a^		0.13 (0.12–0.13)	2177 (2152–2201)
Baseline characteristics			
Sex (ref: female)	Male	0.93 (0.9–0.97)	−81 (−118 to −45)
Systolic blood pressure (centred at 140; per 20 mmHg)		^b^	22 (3–41)
Prior diabetes (ref: no)	Yes	1.11 (1.01–1.22)	^b^
Time-updated characteristics			
Current age (centred at 60; per 10 years)		1.37 (1.34–1.4)	158 (136–179)
Myocardial infarction (ref: no)	Same year	36.83 (24.07–56.37)	3421 (2949–3893)
	1 year ago	2.04 (1.34–3.11)	841 (323–1359)
	2 years ago	1.87 (1.17–2.97)	332 (−125 to 789)
	≥3 years ago	1.34 (1.01–1.77)	372 (87–657)
Stroke (ref: no)	Same year	38.7 (24.72–60.59)	4697 (4059–5335)
	1 year ago	2.87 (1.91–4.31)	1995 (1377–2612)
	2 years ago	2.26 (1.42–3.58)	488 (16–961)
	≥3 years ago	1.62 (1.28–2.05)	924 (635–1213)
Vascular death (ref = no)	Yes	^b^	4786 (2639–6933)
Non-vascular death (ref = no)	Yes	9.56 (7.44–12.29)	4984 (4502–5466)

To predict the annual costs using the two-part model, please follow the following steps

(1) predict the odds of incurring any costs in the year (${\mathrm{Odds}}_{\mathrm{P}1}$) from the first part: ${\mathrm{Odds}}_{\mathrm{P}1}={\mathrm{exp}}^{\mathrm{ln}\left(Intercept\right)+{\sum }_{1}^{n}{(\mathrm{ln}(OR}_{i})*{X}_{i})}$

(2) predict the annual costs assuming such were incurred in the year (${\mathrm{Cost}}_{\mathrm{P}2}$) from the second part: ${\mathrm{Cost}}_{\mathrm{P}2}=Intercept+{\sum }_{1}^{n}{(Mean}_{i}*{X}_{i})$

(3) calculate the predicted annual costs using this formula: ${\mathrm{Odds}}_{\mathrm{P}1}$/(${\mathrm{Odds}}_{\mathrm{P}1}$+1) * ${\mathrm{Cost}}_{\mathrm{P}2}$

Where ${X}_{i}$ is the value of the i^th^ covariate (excluding the intercept term)

*CIs* confidence intervals, *OR* odds ratio

^a^The intercept terms represent the respective values for an individual in the reference categories of the covariates (odds for part 1 model and cost for part 2 model); other coefficients represent the added effect for that category of the covariate compared with the reference category (odds ratio for part 1 model and additional cost for part 2 model)

^b^Covariate was excluded during the selection procedure (not statistically significant)

### Step 4. Use of Developed Model

#### Individual Predictions

**Fig. 4:**
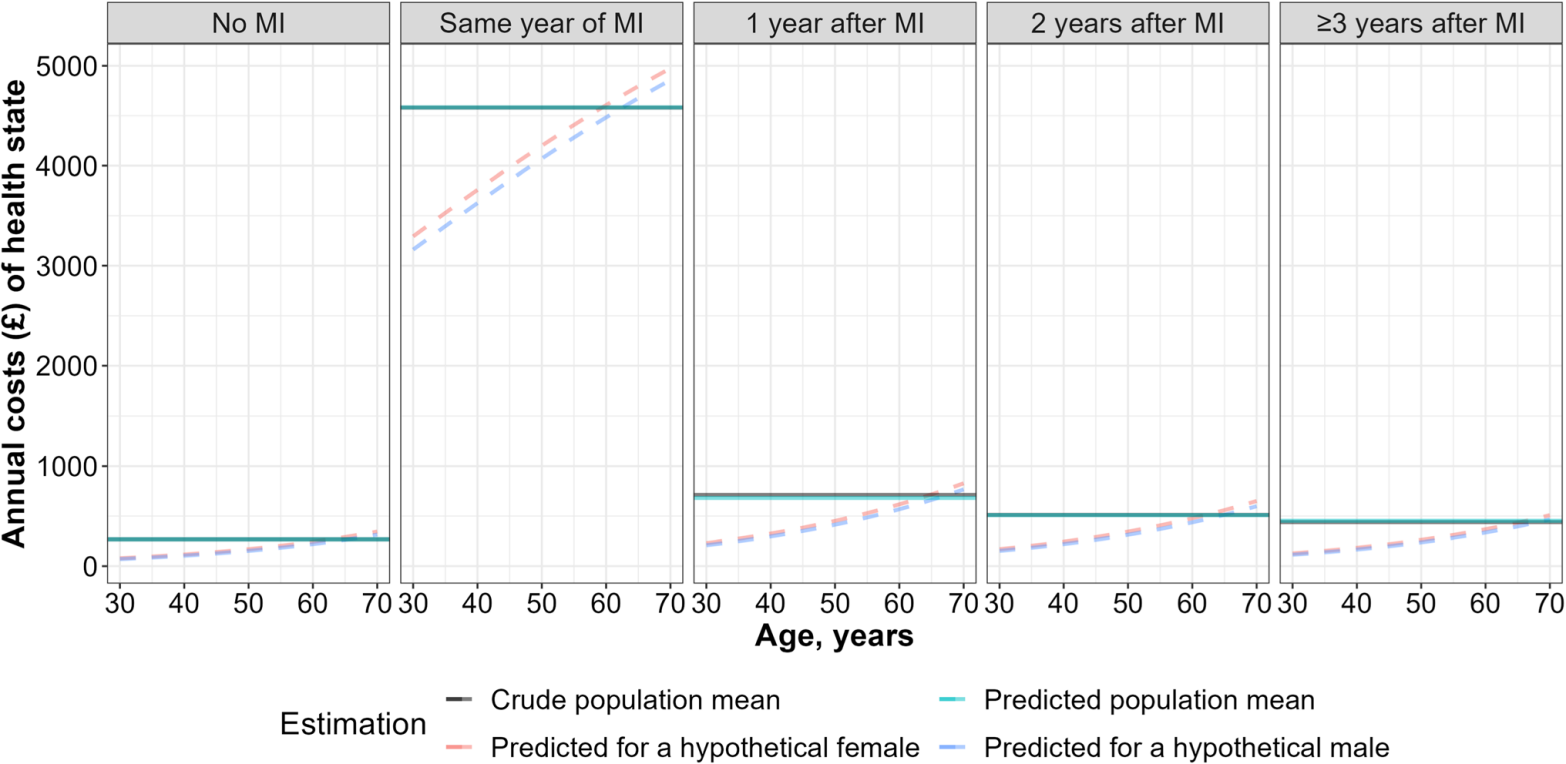
Estimated annual hospital costs associated with MI states using different approaches. *MI *myocardial infarction. Crude population mean: average costs across the population in the disease state;Predicted population mean: average predicted costs across the population in the disease state;Predicted for a hypothetical female/male: predicted costs for the female/male with systolic blood pressure of 140 mmHg, without diabetes, stroke, vascular death or non-vascular death at different ages in the disease state

The developed model can be used for individual predictions. Code S8 presents the annual hospital cost predictions using the final model for an individual with the following profile: a 50-year old female, with systolic blood pressure of 120 mmHg, diagnosed with diabetes mellitus, had a MI in the year, a stroke 1 year ago, without other incident cardiovascular or other events modelled. The predicted probability of incurring any costs in the year was 0.92, and the costs conditional on any incurring totaled 7413; therefore, the predicted annual cost for that year was 6783 (0.92 × 7413). A more detailed illustration of the individual predictions process can be found in the published analyses [8]. Overall, the prediction model resulted in similar estimates of overall average costs for each disease state as the crude average estimate and allowed the impact of age on the costs to be incorporated, which could be extended to also include the other model covariates (e.g. sex, lifestyle factors and previous morbidities) (Fig. 4).

#### Marginal Effect Estimation

Of interest is the marginal effect of the disease state, that is, the excess costs associated with a particular (temporal history of) disease/event compared with no disease/event. The developed model can be used to estimate the mean marginal effect of the disease state on costs. We firstly estimated the mean effect by recycled prediction. For example, to estimate the marginal effect of ‘Had MI in same year’ on annual hospital costs, we (1) modified the analytical dataset, setting the covariate of ‘MI’ to (a) ‘Had MI in same year’ and then (b) ‘None’ in turn for each of the annual periods to create two versions of the dataset; (2) predicted the costs with each modified dataset and (3) calculated the mean difference between the two predicted costs. The mean difference was the marginal effect of the ‘Had MI in same year’. The standard error of the marginal effect was estimated using 1000 bootstrapped samples. For the population represented by the synthetic analytical dataset, the marginal effect of the ‘Had MI in same year’ on annual hospital care costs (£, 95% confidence intervals) was 4326 (3801–4851). The marginal effect for the other disease states was also estimated (Table 5) (Code S9).

**Table 5:** Excess annual hospital care costs (£) associated with cardiovascular events and non-vascular death

Event (Ref = no)	Year since event	Marginal effect (95% CIs)
Myocardial infarction	Same year	4326 (3801–4851)
	1 year ago	382 (149–615)
	2 years ago	240 (34–446)
	≥ 3 years ago	128 (28–228)
Stroke	Same year	5417 (4749–6085)
	1 year ago	876 (515–1237)
	2 years ago	353 (106–600)
	≥ 3 years ago	290 (170–410)
Vascular death	Yes	559 (247–871)
Non-vascular death	Yes	3658 (3154–4162)

*CIs* confidence intervals

## Discussion

### Summary

In this tutorial, we provided a step-by-step guide to modelling healthcare costs associated with disease states with an illustrative example of modelling cardiovascular disease costs from a published study. We presented the detailed process and practical illustration of such modelling after the conceptualization of the research question, which includes the analytical dataset preparation, detailed model development and utilization of the developed model. The process we used addressed the issues related to the nature of costing data, with lots of zero observations and highly right skewed distribution among the non-zero observation, and was relatively easy to implement and interpret. Although the illustrative example applied such modelling only in the context of cardiovascular disease, the approach is general and can be applied to any disease area [5, 7, 25].

### Further Remarks

In this tutorial we presented a general approach to estimating costs of disease states using individual patient data. It is good research practice to pre-specify many of the elements of these analyses prior to conducting the analysis to minimize potential biases [26]. These include the disease state indicators, individual patient characteristics, types of statistical models to consider and the approaches to selection of covariates. In the illustrative example, we used stepwise backward elimination for covariate selection, a simple, widely available and still widely used approach. However, the stepwise approach may result in selection of unstable predictors. We illustrated one approach of addressing this instability using the bootstrapping stepwise selection approach with high cut-off criteria but other approaches for covariate selection may be considered [15].

### Advantage of the Tutorial

Our tutorial provides a general approach to developing healthcare cost models using individual patient data, which is frequently called upon in the field of health economic evaluation. Although a number of cost models have been reported, the rationale behind the modelling process is usually not fully explained. In this tutorial, based on our experience [1, 5–7], we propose a number of steps researchers can employ to justify their choices. Our illustrative example takes the users through the practicalities of implementing the steps in R, which fills a gap in this area.

### Limitation of Tutorial

We only listed a few frequently used options at each modelling step (e.g. statistical model choice, covariate selection, model selection), but they are not exhaustive sets of options. Therefore, this tutorial could be considered an introduction into cost modelling. To avoid distracting the readers, we did not discuss in detail alternative methods at different stages of the modelling process. Instead, we provided key references comparing these methods for readers to explore further. The use of the UK Biobank dataset requires a specific application process, and the dataset cannot be shared externally. To enable sharing of our workings in the illustrative example, we used a synthetic dataset. Therefore, the estimated relationships in this tutorial are for illustration purpose only. Interested users in models of costs associated with cardiovascular events can refer to our original study on this topic [8]. In this tutorial we also do not illustrate the approaches to deal with missing data or functional form of continuous covariates, which are dealt with elsewhere [16]. The illustrative example, however, shows all the key stages of cost modelling using individual patient data to inform economic evaluation.

## Conclusion

To our knowledge, this is the first tutorial for modelling healthcare costs associated with disease states in decision analytic models using individual patient data. We hope it is a useful starting point for researchers who plan to conduct such an analysis.

## Supplementary Information

Below is the link to the electronic supplementary material.

Supplementary file1 (DOCX 41 KB)

Supplementary file2 (ZIP 6 KB)

## Declarations

### Data availability statement

All needed data and materials are presented in the manuscript or the supporting information. No extra data will be needed. Software code for implementing the illustrative example using R is provided.
