# Supplementary material for: Estimating Costs Associated with Disease Model States Using Generalized Linear Models: A Tutorial
Source: Pharmacoeconomics. 2023 Nov 10;42(3):261–73. doi: 10.1007/s40273-023-01319-x (PMC11424740; doi:10.1007/s40273-023-01319-x)
Supplement: Supplementary file 1 — Supplementary file1 (DOCX 41 KB) [file 40273_2023_1319_MOESM1_ESM.docx]

**Estimating Costs Associated with Disease Model States Using Generalized Linear Models: A Tutorial**

*PharmacoEconomics*

Zhou J, Williams C, Keng MJ, Wu R, Mihaylova B

**Corresponding to:** Dr. Junwen Zhou, Health Economics Research Centre, Nuffield Department of Population Health, Old Road Campus, Headington, Oxford, UK. email: junwen.zhou@ndph.ox.ac.uk.

## Supplementary section 1. Simulation of the synthetic data for the illustrative example.

We simulated synthetic data for 10,000 participants with 10 annual periods for each participant using the published cost model^1^. Firstly, we simulated each baseline covariate of participants independently based on the data type of the covariate: continuous covariates were simulated using the normal distribution based on the population mean and standard deviance; binary covariates were simulated using the Bernoulli distribution based on the population proportion; and categorical covariates were simulated using a generalized Bernoulli distribution based on the population proportion for each category. The year of occurrence of disease for each disease of interest was simulated using the exponential distribution based on the population annual rate. Finally, costs for each annual period were simulated based on a simplified version of the published two-part models^1^, with the occurrence of costs in the annual period sampled from a Bernoulli distribution and the annual costs conditional on any occurring sampled from the Normal distribution. (Table SS1, Code S1)

**Table SS1. Parameters for the simulation of the synthetic dataset**

| **Parameter name** | **Category** | **Parameter value** | |
| --- | --- | --- | --- |
| **Baseline covariates (N = 444 536)** | | **Mean/N** | **SD/Proportion** |
| Age (years) |  | 56.0 | 8.1 |
| Male | Yes | 194 979 | 43.9 |
| Ethnicity | White | 417 964 | 94 |
|  | Black | 7 266 | 1.6 |
|  | South Asian | 6 983 | 1.6 |
|  | Other* | 9 912 | 2.2 |
|  | Missing | 2 411 | 0.5 |
| Townsend socioeconomic deprivation | Quintile 1  (least deprived) | 166 039 | 37.4 |
|  | Quintile 2 | 89 211 | 20.1 |
|  | Quintile 3 | 72 492 | 16.3 |
|  | Quintile 4 | 64 358 | 14.5 |
|  | Quintile 5 | 51 883 | 11.7 |
|  | Missing | 553 | 0.1 |
| Smoking | Never | 248 296 | 55.9 |
|  | Former smoker | 147 781 | 33.2 |
|  | Current smoker | 45 979 | 10.3 |
|  | Missing | 2 480 | 0.6 |
| Physical activity | Low | 65 921 | 14.8 |
|  | Moderate | 146 146 | 32.9 |
|  | High | 145 192 | 32.7 |
|  | Missing | 87 277 | 19.6 |
| Diet quality | Healthy | 285 989 | 64.3 |
|  | Unhealthy | 149 077 | 33.5 |
|  | Missing | 9 470 | 2.1 |
| Body mass index (kg/m^2^) | <18.5 | 2 364 | 0.5 |
|  | ≥18.5, <25 | 148 846 | 33.5 |
|  | ≥25, <30 | 187 957 | 42.3 |
|  | ≥30, <35 | 74 396 | 16.7 |
|  | ≥35, <40 | 20 645 | 4.6 |
|  | ≥40 | 7 871 | 1.8 |
|  | Missing | 2 457 | 0.6 |
| LDL cholesterol (mmol/L) |  | 3.6 | 0.8 |
| HDL cholesterol (mmol/L) |  | 1.5 | 0.4 |
| Creatinine (umol/L) |  | 71.5 | 15.1 |
| Systolic blood pressure (mmHg) |  | 137.8 | 18.6 |
| Diastolic blood pressure (mmHg) |  | 82.4 | 10.1 |
| On antihypertensive treatment | Yes | 71 925 | 16.2 |
| Prior diabetes | Yes | 21 562 | 4.9 |
| Prior cancer | Yes | 32 712 | 7.4 |
| Severe mental illness history | Yes | 36 082 | 8.1 |
| **Incident event (7.1 years follow-up)** | | **N** | **Proportion** |
| Myocardial infarction | Yes | 4 651 | 1.0 |
| Stroke | Yes | 4 106 | 0.9 |
| Vascular death | Yes | 1 781 | 0.4 |
| Non-vascular death | Yes | 9 067 | 2.0 |
| **Simplified two-part model of annual hospital care costs** | | **Part 1: odd of any costs**  **(Odd ratios)** | **Part 2: cost, if any incurred**  **(Mean difference)** |
| Intercept |  | 0.13 | 2102 |
| Sex (Ref: female) | Male | 0.92 | -65 |
| Current age (centre at 60; per 10 years) |  | 1.38 | 173 |
| Myocardial infarction (Ref: No) | Same year | 47.09 | 3 054 |
|  | 1 year ago | 1.76 | 670 |
|  | 2 years ago | 1.44 | 304 |
|  | ≥3 years ago | 1.35 | 304 |
| Stroke (Ref: No) | Same year | 47.08 | 4 485 |
|  | 1 year ago | 2.58 | 2 192 |
|  | 2 years ago | 1.78 | 833 |
|  | ≥3 years ago | 1.49 | 833 |
| Vascular death (Ref: No) | Yes | 2.32 | 4 318 |
| Non-vascular death (Ref: No) | Yes | 11.4 | 6 792 |

HDL, high density lipoprotein; LDL, low density lipoprotein; SD, standard deviation.

## Supplementary table 1. Baseline covariates specification from the raw dataset

| **Covariates** | **Raw dataset** | | | **Analytical dataset** | |
| --- | --- | --- | --- | --- | --- |
|  | **Specification** | | **Values** | **New specification** | **Values** |
| **Age (years)** | Z | 56 (8) | | (Z – 60)/10 | -0.4 (0.8) |
| **Sex** | Female | 5635 (56.4) | | Female* | - |
|  | Male | 4365 (43.6) | | Male | - |
| **Ethnicity** | White | 9464 (94.6) | | White* | - |
|  | Black | 179 (1.8) | | Black | - |
|  | South Asian | 165 (1.7) | | South Asian | - |
|  | Others | 192 (1.9) | | Others | - |
| **Townsend deprivation score, categorized into quintiles**  **(Quintile 1: least deprived)** | Quintile 1 | 3712 (37.1) | | Quintile 1 | - |
|  | Quintile 2 | 1947 (19.5) | | Quintile 2 | - |
|  | Quintile 3 | 1701 (17) | | Quintile 3* | - |
|  | Quintile 4 | 1449 (14.5) | | Quintile 4 | - |
|  | Quintile 5 | 1191 (11.9) | | Quintile 5 | - |
| **Smoking** | Never | 5584 (55.8) | | Never* | - |
|  | Former | 3365 (33.7) | | Former | - |
|  | Current | 1051 (10.5) | | Current | - |
| **Physical activity** | Low | 1807 (18.1) | | Low | - |
|  | Moderate | 4081 (40.8) | | Moderate* | - |
|  | High | 4112 (41.1) | | High | - |
| **Diet quality** | Healthy | 6581 (65.8) | | Healthy* | - |
|  | Unhealthy | 3419 (34.2) | | Unhealthy | - |
| **Body mass index (BMI, kg/m^2^), categorized** | <18.5 | 53 (0.5) | | <18.5 | - |
|  | ≥18.5, <25 | 3295 (33) | | ≥18.5, <25* | - |
|  | ≥25, <30 | 4334 (43.3) | | ≥25, <30 | - |
|  | ≥30, <35 | 1682 (16.8) | | ≥30, <35 | - |
|  | ≥35, <40 | 449 (4.5) | | ≥35, <40 | - |
|  | ≥40 | 187 (1.9) | | ≥40 | - |
| **LDL cholesterol (mmol/L)** | Z | 3.6 (0.8) | | (Z - 3.6) / 1 | 0 (0.8) |
| **HDL cholesterol (mmol/L)** | Z | 1.5 (0.4) | | Ln(Z) | 0.4 (0.3) |
| **Creatinine (umol/L)** | Z | 71.7 (15.2) | | (Ln(Z) - 4.4)/ 0.2 | -0.8 (1.1) |
| **Systolic blood pressure (mmHg)** | Z | 137.7 (18.7) | | (Z – 140) /20 | -0.1 (0.9) |
| **Diastolic blood pressure (mmHg)** | Z | 82.3 (10) | | (Z – 80) / 10 | 0.2 (1) |
| **On antihypertensive treatment** | None | 8416 (84.2) | | None* | - |
|  | Yes | 1584 (15.8) | | Yes | - |
| **Prior diabetes** | None | 9503 (95) | | None* | - |
|  | Yes | 497 (5) | | Yes | - |
| **Prior cancer** | None | 9262 (92.6) | | None* | - |
|  | Yes | 738 (7.4) | | Yes | - |
| **Severe mental illness** | None | 9172 (91.7) | | None* | - |
|  | Yes | 828 (8.3) | | Yes | - |

Values are mean (SD) or number (%).

HDL, high density lipoprotein; LDL, low density lipoprotein;

Z: value with the same unit as shown in the candidate continuous covariates;

-: the same as the value shown in the former step;

*: reference level for the binary/categorical covariate;

## Supplementary table 2. Illustration of instability of selection results from stepwise approach for Gamma-Identity GLM costs model conditional on any incurred costs

| Covariate | Proportion of being included in 100 bootstrap samples using bootstrapping stepwise backward elimination^a^ | Being included in the original sample using stepwise backward elimination | True predictor from data generation (supplementary section 1) |
| --- | --- | --- | --- |
| Age | 1.00 | YES | YES |
| Sex | 1.00 | YES | YES |
| MI | 1.00 | YES | YES |
| NVD | 1.00 | YES | YES |
| Stroke | 1.00 | YES | YES |
| VD | 0.97 | YES | YES |
| Body mass index | 0.60 | NO | NO |
| Systolic blood pressure | 0.56 | YES^b^ | NO |
| Ethnicity | 0.52 | NO | NO |
| Prior cancer | 0.39 | NO | NO |
| Physical activity | 0.38 | NO | NO |
| HDL cholesterol | 0.17 | NO | NO |
| Townsend score | 0.15 | NO | NO |
| Smoking status | 0.13 | NO | NO |
| Prior diabetes | 0.11 | NO | NO |
| Antihypertensive treated | 0.08 | NO | NO |
| Serum creatinine | 0.07 | NO | NO |
| LDL cholesterol | 0.05 | NO | NO |
| Severe mental illness | 0.05 | NO | NO |
| Diet quality | 0.05 | NO | NO |
| Diastolic blood pressure | 0.03 | NO | NO |

GLM: generalized linear model; HDL: high density lipoprotein; LDL: low density lipoprotein; MI: myocardial infarction; NVD: non-vascular death; VD: vascular death;

^a^: Usually more than 1000 bootstrap samples should be used. We chose less samples for the sake of computational burden, as 100 bootstrapping approach already took 1 hour to run.

^b^: The nuisance predictor included by the stepwise approach.

## **Supplementary materials reference**

1. Zhou J, Wu R, Williams C, et al. Prediction Models for Individual-Level Healthcare Costs Associated with Cardiovascular Events in the UK. Pharmacoeconomics. 2023. doi: 10.1007/s40273-022-01219-6.
